# Prevalence of emotional and behavioral problems and subthreshold psychiatric disorders in Austrian adolescents and the need for prevention

**DOI:** 10.1007/s00127-018-1586-y

**Published:** 2018-08-29

**Authors:** Julia Philipp, Michael Zeiler, Karin Waldherr, Stefanie Truttmann, Wolfgang Dür, Andreas F. K. Karwautz, Gudrun Wagner

**Affiliations:** 1 Department for Child and Adolescent Psychiatry, Medical University of Vienna, Waehringer Guertel 18-20, 1090 Vienna, Austria; 2 FernFH Distance Learning University of Applied Sciences, Zulingerstraße 4, 2700 Wiener Neustadt, Austria; 3 Institute of Sociology, University of Vienna, Rooseveltplatz 2, 1090 Vienna, Austria

**Keywords:** Mental health, Emotional and behavioral problems, Epidemiology, Adolescents, Subthreshold disorders

## Abstract

**Purpose:**

Epidemiological data are crucial to plan adequate prevention strategies. Thus, this study aims at obtaining the prevalence of mental health problems (MHP) and subthreshold psychiatric disorders based on a representative sample of Austrian adolescents.

**Methods:**

Adolescents aged 10–18 were recruited from Austrian schools. Emotional and behavioral problems were determined using the Youth Self-Report (YSR); the point prevalence of subthreshold psychiatric disorders was assessed using structured diagnostic interviews. Sociodemographic variables including socioeconomic background, migration status, family structure, and place of residence were obtained. In addition, a non-school sample (unemployed adolescents, and child and adolescent psychiatry patients) was included to enhance representativeness and generalizability.

**Results:**

3446 students, 37 unemployed adolescents, and 125 child and adolescent psychiatric patients provided analyzable YSR data sets. In the school sample, 16.5% scored in the clinically relevant range, while internalizing problems were more prevalent (17.8%) than externalizing problems (7.4%). These prevalences increased by 0.7–2.0% when the non-school sample was taken into account. A low socioeconomic status (SES) and living in single parent families were associated with higher problem scores. Regarding the interviewed sample (377 students and 407 parents), subthreshold psychiatric disorders were observed in 12.7% of students. 92.5% of them have not yet received any kind of help.

**Conclusions:**

A significant proportion of Austrian adolescents are at risk for MHP. A non-responder analysis indicates that the observed prevalence may be even underestimated. These findings emphasize the urgent need for targeted prevention, especially for reducing anxiety and depressive symptoms and for adolescents in disadvantaged families.

## Introduction

Mental health problems (MHP) in children and adolescents are of substantial importance in public health. Epidemiological studies offer essential information about prevalence and correlates of MHP and full-syndrome disorders, serve as basic precondition for mental health policy like prevention and treatment strategies, and are important for the evaluation of existing and the planning of future mental health services [1–3].

Several studies obtained the prevalence of full-syndrome psychiatric disorders among adolescents. Ihle and Esser [2] found a mean period prevalence of 18% (6.8–37.4%) in their meta-analysis between 1970 and 2000. A recent meta-analysis found a world-wide pooled-prevalence of 13.4% for any disorder [4]. Another review of US and Great Britain samples between 2000 and 2007 revealed that 25% of children and adolescents suffer from a psychiatric disorder during the past year and one out of three during their whole life [5]. In Austria, a point prevalence of 23.9% was found for any full-syndrome psychiatric disorder using DSM-5 criteria [6].

Prevalence figures of full-syndrome disorders are important to plan suitable treatment strategies. However, a detailed description of the population at risk for psychiatric disorders is of additional importance as this group should be approached to prevent symptom progression [7]. This is especially important given that adolescent MHP tend to persist into adulthood [1, 2, 8] and adult MHP often begin in adolescence [1, 8–11]. In the German BELLA study, 21.9% of children and adolescents showed MHP [12]. Steinhausen [13] reported that one-third of affected adolescents develop a psychiatric disorder in adulthood; Ravens-Sieberer et al. [14] reported 10% persistent or new MHP after 6 year follow-up.

Persistent MHP increase the risk for further societal and individual problems. Psychiatric disorders play a leading role concerning costs for the health care system [15], with five out of ten leading causes of disability-adjusted life years being psychiatric disorders. MHP lead to high burden and result in lower educational achievements, violence, substance abuse, and poor reproductive and sexual health [11]. MHP in childhood and adolescence increase the risk for sickness absence and disability pension in young adulthood [16]. Furthermore, quality of life is reduced [12, 17].

Data on subthreshold disorders in adolescents are not yet presented for a large population, although there are several articles focusing on single disorders such as depression or eating disorders. Approximately 2–29% [18, 19] of adolescents suffer from subthreshold depression; 11–15% suffer from subthreshold eating disorders [20, 21]. There is evidence that subthreshold depression causes impairment by negatively effecting Quality of Life [18] causing negative consequences concerning psychosocial functioning, social relations, and school performance [22] in adolescence and leading to an elevated risk of subsequent depression and suicidal behavior [23]. These negative effects are comparable to the effects of full-syndrome depression [18, 22, 23], implicating that subclinical cases should rather be treated like full-syndrome cases than like healthy controls [23].

In longitudinal studies, half of the cases with subthreshold depression showed an increase in depressive symptoms [24] and one-third of subclinical cases developed full-syndrome depression within 12 months [25]. Prevention can hamper up to one quarter of new cases of depression cases per year or at least delay the onset of clinically relevant symptoms [26] and is known to have a positive impact on subclinical symptoms [27].

Therefore, addressing the population at risk and those with subthreshold disorders and implementing suitable targeted prevention strategies are of great importance, whereas universal prevention is directed at all adolescents in a defined setting to reduce general risk; targeted prevention is directed at either high-risk groups (selective prevention) or adolescents with subclinical symptoms (indicated prevention) to prevent full-syndrome disorders [7, 22, 26, 28]. All of them have been found to effectively reduce MHP in children and adolescents [28], but prevention programs targeted at high-risk populations generally show higher effects than universal prevention programs [22, 26, 27]. As the early prevention can improve the well-being and productivity, decrease future burden, provide health and socioeconomic benefits, reduce adult psychiatric disorders, and reduce costs for the healthcare system, addressing MHP in adolescents is a priority for the global health agenda due to ethical responsibility and economic reasons [28].

Research on sociodemographic correlates of MHP is of special interest to identify high-risk groups who should be considered specifically for indicated prevention strategies [28]. In general, boys seem to have higher rates of 12-month disorders than girls [10]. Girls more often suffer from internalizing, boys from externalizing problems [2, 29]. Older adolescents report more total [29] and internalizing problems [1], and more mood and substance use problems [10]. In childhood, boys have more MHP than girls. In adolescence, the prevalence rates are similar [1, 2] or even higher for girls [8]. Low socioeconomic status (SES) was mostly related to an increased prevalence of MHP [9, 11, 12, 30, 31]. Some studies suggest a negative effect of migration [31–33]; other studies show inconsistent results, no differences, or even lower rates of MHP in migrants [34, 35]. Adolescents living with both parents report lower rates of MHP [9, 10, 31, 36, 37]. Some findings point out higher rates of MHP for urban than rural areas [9, 31], another study reported no such association [10].

The Mental Health in Austrian Teenagers (MHAT) Study is the first large epidemiological study collecting data on mental health in a representative national sample of adolescents aged 10–18 years. Besides the prevalence of full-syndrome DSM-5 disorders [6], this study aims to obtain the prevalence of emotional and behavioral problems and subthreshold psychiatric disorders as well as sociodemographic correlates (SES, migration background, familial constellation, and region).

## Methods

### Sample selection, recruitment, and procedure

All secondary schools in Austria (*N* = 2547) were asked to participate. Of those willing to take part, a representative random cluster sample of classes stratified by grade, school type, and federal state was selected. Four school grades (5th, 7th, 9th, and 11th) were included. Adolescents and legal representatives provided informed consent prior to inclusion in the study. Adolescents with low German language skills were excluded. Adolescents attending a school for mentally handicapped were also excluded.

Data collection and obtainment of written informed consents were organized by teachers. Questionnaires were available as online or paper–pencil version and were administered during a school lesson (approximately 50 min). The feasibility and acceptability of the procedure for teachers and the feasibility of the online-application were confirmed in a pilot study [38].

As several findings point out an underestimation of the prevalence of self-reported health variables due to non-response [39, 40], a non-responder analysis was additionally conducted to evaluate a potential non-response bias.

To enhance representativeness and generalizability of results, a non-school sample was also included. This procedure for epidemiological studies was suggested by Barkmann and Schulte-Markwort [41] as prevalence rates tend to be underestimated using a school sample only. Two subsamples were additionally included: First, the early school leavers were recruited from courses for unemployed adolescents. Course providers were selected randomly and asked for participation. Four course providers (two in urban and two in rural areas of Austria) were selected and course participants were approached. The same procedure of data collection as in the school sample was applied with the exception that the paper–pencil version of the questionnaire was used only. Second, adolescents who cannot attend a regular school due to severe MHP were recruited from child and adolescent psychiatry wards. Eight wards in five federal states of Austria were included and all inpatient adolescents from 10 to 18 years were asked to participate.

In a second stage, all adolescents detected to be at risk for MHP as well as a random sample of adolescents not at risk and one parent each were invited to take part in a structured telephone interview to obtain DSM-5 diagnoses. We defined adolescents to be “at risk” when they scored above the clinical threshold in at least one syndrome scale of the YSR or in an extra-screening instrument for eating disorders (SCOFF, [42]). Details on the sample selection and procedure are published elsewhere [6, 43, 44].

### Sample size calculation

Sample size calculation is based on a cluster sample using the procedure suggested by Sullivan [45]. In accordance with the previous epidemiological studies, a prevalence of 20% [1, 2] was assumed. A number of 60 clusters were intended per school grade and a design effect of 2 was assumed. The calculated sample size was 502 per school grade and sex leading to a total planned sample size of 4016. A certain proportion cannot be reached via the school setting due to the early school leaving or severe mental health conditions. This proportion is based on the national report of education [46] and known figures about the proportion of adolescents in treatment [47], resulting in 40 adolescents to be recruited from courses for unemployed adolescents and 128 adolescents to be recruited from child and adolescent psychiatry wards.

### Instruments

#### Emotional and behavioral problems

The Youth Self-Report (YSR) [48] (German version: [49]), a widely used questionnaire [29, 50, 51] to assess emotional and behavioral problems, was used to obtain the prevalence of adolescents at risk for MHP. The 103 problem behaviors are rated on a three-point scale. Items are summed up to eight syndrome scales (withdrawn, somatic complaints, anxious/depressed, social problems, thought problems, attention problems, delinquent behavior, and aggressive behavior) and three broadband scales (total problem score, internalizing problems, and externalizing problems). Higher scores indicate more problems. For the broadband scales, good internal consistencies are reported (*α* > 0.86), for the syndrome scales, Cronbach’s α is between 0.56 and 0.86. Raw scores are transferred into *T* scores using German norm data. According to the manual, the established cutoffs (*T* > 63 for broadband scales, *T* > 69 for syndrome scales) are used to define at-risk cases. DSM-oriented scales of the YSR have not been available in German when the study started [52, 53]. Cross-informant agreement between the Youth Self-Report and the correspondent Child Behavior Checklist (CBCL, [53]) assessing mental health problems from the parents’ view is not very high (*r* = 0.41 [50]). Therefore, we refrained from involving the CBCL also in the screening phase. As YSR mean scores are known to be significantly higher than CBCL mean scores for all problem scales [50], only adolescent`s rating was included in this study to easier detect the proportion of participants at risk.

#### Sociodemographic variables

An extended version of the Family Affluence Scale (FAS, [54]) was used to assess the SES of the family. The FAS is a self-report measurement developed for children and adolescents consisting of four items (number of cars in the family, having a room of one’s own, amount of holidays spent, and number of family owned computers) which are rated on a 2–4-point scale. For this study, two additional items were obtained (having a dishwasher and number of bathrooms) which were used in the latest survey of the Health Behavior in School-Aged Children Study [55]. Item ratings are summed up, higher scores indicating a higher level of family affluence. For the purpose of this study, low, medium, and high SES were defined as scoring below the 25th percentile (low), between the 25th and the 75th percentile (medium) and above the 75th percentile (high).

Migration background was coded when at least the adolescent or one parent was born in a foreign country. Family constellation was obtained by asking with whom the adolescent lives with. Answers were coded into three categories: living with both biological parents, living with a single parent, and living in a patchwork family. Place of residence was divided into rural areas (< 10.000 inhabitants) and urban areas (> 10.000 inhabitants).

#### Teacher’s questionnaire

Teachers were asked to anonymously provide information on a few basic variables that can indicate MHP in students including sex, repetition of classes (yes/no), school absenteeism, willingness to make an effort, ability to concentrate (more/less/similar than other students), social integration in class, passivity, disciplinary problems (rather yes/no) and parental contact made due to behavioral problems (yes/no/no, but should be done). Both participating and non-participating students were rated.

#### Subthreshold DSM-5 psychiatric disorders

Adolescents who were screened at risk for MHP as well as a random sample of students not at risk and one parent were invited for a structured clinical telephone interview conducted by clinical psychologists. The Childrens’ Diagnostic Interview for Mental Disorders (CDI-MD; [56]) that was adapted for DSM-5 diagnostic criteria was used to obtain a total of 35 disorders from nine diagnostic groups. To minimalize participants’ burden, the different disorders were divided between the adolescent’s and the parent’s interview. Internalizing psychiatric disorders were only assessed in the adolescents’ interview, whereas externalizing disorders and disorders that mainly occur in infancy and early childhood were only assessed in the parents’ interview, as internalizing problems are more validly assessed by adolescents themselves and externalizing problems by parents [56]. The prevalence figures of full-syndrome disorders were recently published [6]. In addition, the current subthreshold diagnoses were obtained. Subthreshold diagnoses were given when one diagnostic criterion was not met and the diagnosis did not fall into the residual category “other specified N.N. disorder”.

#### Health-related quality of life (HrQoL)

The KIDSCREEN questionnaire [57] was used to obtain aspects of HrQoL including a global measure of HrQoL, self-perception, parent relation and home life, social support and peers, school environment, and bullying. This measure can serve as a proxy for psychosocial impairment in different domains. Items are rated on a 5-point Likert scale. Internal consistencies were good for all used dimensions (Cronbach Alphas 0.77–0.89). Raw scores are transferred into *T* Scores according to the available German normative data. A low HrQoL is defined as scoring below the 25th quantile. In addition, adolescents rated their school performance on a 4-point scale (very good to below average). Furthermore, parents were asked if there were any problems in school, with family and peers and conflicts with the law.

### Data analyses

For prevalence calculations, weighting procedures were applied to adjust for deviations from the original sample plan due to non-response. All cases were weighted with the inverse proportion of obtained number of cases related to the planned number of cases in one group (school type × school grade × federal state). As there were a high number of clusters and a low number of individuals within the clusters, the design effect was lower than expected (about 1). Thus, we refrained from calculating prevalence estimates that are corrected for cluster sampling.

In a second step, prevalence estimates were also calculated for the non-school sample (early school leavers and inpatients). Finally, the prevalence estimates of the school and non-sample were pooled to obtain a total prevalence that takes into account that a certain proportion of adolescents cannot be reached via the school setting. School and non-school samples were weighted in accordance to the original sample plan.

Comorbid MHP were analyzed by calculating how many percent of adolescents scoring in the clinical range of one YSR syndrome scale reach the cutoff in at least one other syndrome scale. Logistic regression analyses and odds ratios were used to analyze sociodemographic predictors (SES, migration background, region, and family constellation) of clinically relevant YSR scores. For the non-responder analysis, logistic regression analyses were calculated to predict non-response by variables obtained in the teacher’s questionnaire.

Prevalence estimates of the subthreshold DSM-5 disorders in the school sample were calculated applying the law of total probability. The prevalence estimates within the sample screened at risk and the sample screened not at risk were pooled by weighting them with the probability for being at risk, respectively, not at risk.

## Results

### Sample characteristics

Of all secondary schools in Austria (*N* = 2547), 428 (16.8%) agreed to participate. A stratified random sample of classes was drawn. Of the 7643 adolescents approached, 3615 (47.3%) gave informed consent and 3446 (45.1%) produced analyzable data and were, therefore, included in the analysis. Sociodemographic characteristics of the school sample are shown in Table 1.

**Table 1:** Sociodemographic information of the school sample

	*N*	%
Total sample	3446	100
Sex		
Males	1540	44.7
Females	1906	55.3
School grade		
5th	527	15.3
7th	875	24.4
9th	1090	31.6
11th	954	27.7
Socioeconomic status^a^		
Low	772	22.4
Medium	1767	51.3
High	790	22.9
Unknown	117	3.4
Family constellation^b^		
Both biological parents	2477	71.9
Single parent	554	16.1
Patchwork	305	8.9
Unknown	110	3.2
Region^c^		
Urban	1981	57.5
Rural	1418	41.1
Unknown	47	1.4
Migration background^d^		
No	2519	73.1
Yes	866	25.1
Unknown	61	1.8

^a^Based on Family Affluence Scale II (score range: 0–13); categories based on percentiles: low (< 25. perc.), medium (25. < perc. < 75. perc.), and high (> 75. perc.)

^b^In which type of family that the adolescent lives

^c^Urban area (> 10.000 inhabitants); rural area (< 10.000 inhabitants)

^d^Migration background defined as follows: either adolescents or a parent born in a foreign country

Eight child and adolescent psychiatry wards in Austria were approached and agreed to participate. Of 292 inpatient patients, 133 provided informed consent (45.5%) and 125 provided analyzable data sets. Of seven course providers offering courses for unemployed adolescents, four agreed to support the study. Of 76 adolescents who were asked to participate, 43 (56.6%) provided informed consent and 37 provided analyzable data sets.

In total, 377 adolescents and 407 parents of the school sample participated in the interview stage.

### Emotional and behavioral problems in the school sample

In total, 16.5% reported signs of MHP using the YSR total problem score. Clinically relevant internalizing problems were reported more often (17.8%) than externalizing problems (7.4%). Within the syndrome scales of the YSR, somatic complaints, thought problems, social withdrawal, and anxious/depressed were most prevalent, whereas aggressive behavior and delinquent behavior were least frequently mentioned. Girls reported more total problems, internalizing problems, and problems on all second-order scales except somatic complaints, social and thought problems. For externalizing problems, no difference between boys and girls was found. However, externalizing problems seem to rise with increasing age for girls and even more for boys. There were no differences concerning total and internalizing problems between the age groups. All prevalence estimates of the school sample including 95% confidence intervals are depicted in Table 2. The mean total problem score (across both sexes and all school grades) was 34.2 [95% CI: 33.5; 34.9].

**Table 2:** Prevalence (% and 95% confidence intervals) of mental health problems among students (*n* = 3446) obtained by the Youth Self-Report (YSR)

YSR domain	Total sample	Girls					Boys				
		Total girls	5th grade	7th grade	9th grade	11th grade	Total boys	5th grade	7th grade	9th grade	11th grade
Total problems	16.5 [15.3; 17.7]	18.8 [17.0; 20.6]	17.4 [12.7; 22.1]	18.4 [14.8; 22.0]	20.0 [16.9; 23.1]	19.0 [15.8; 22.2]	13.7 [12.0; 15.4]	12.3 [8.4; 16.2]	14.1 [10.8; 17.4]	13.1 [10.0; 16.2]	15.7 [12.0; 19.4]
Internalizing problems	17.8 [16.5; 19.1]	19.6 [17.8; 21.4]	18.2 [13.5; 22.9]	19.8 [16.1; 23.5]	20.7 [17.6; 23.8]	19.6 [16.3; 22.9]	15.5 [13.7; 17.3]	16.6 [12.2; 21.0]	15.7 [12.3; 19.1]	15.3 [12.0; 18.6]	14.0 [10.5; 17.5]
Externalizing problems	7.4 [6.5; 8.3]	7.8 [6.6; 9.0]	1.4 [0.0; 2.8]	8.5 [5.9; 11.1]	10.8 [8.4; 13.2]	9.6 [7.2; 12.0]	6.8 [5.5; 8.1]	3.8 [1.5; 6.1]	5.5 [3.4; 7.6]	7.2 [4.8; 9.6]	11.9 [8.6; 15.2]
Social withdrawal	4.3 [3.6; 5.0]	5.1 [4.1; 6.1]	6.1 [3.2; 9.0]	2.4 [1.0; 3.8]	6.2 [4.3; 8.1]	5.6 [3.7; 7.5]	3.4 [2.5; 4.3]	1.7 [0.2; 3.2]	3.9 [2.1; 5.7]	3.8 [2.0; 5.6]	4.4 [2.3; 6.5]
Somatic complaints	6.8 [6.0; 7.6]	6.6 [5.5; 7.7]	6.9 [3.8; 10.0]	4.4 [2.5; 6.3]	6.8 [4.8; 8.8]	8.0 [5.8; 10.2]	7.2 [5.9; 8.5]	9.7 [6.2; 13.2]	7.6 [5.1; 10.1]	4.4 [2.5; 6.3]	6.2 [3.8; 8.6]
Anxious/depressed	4.8 [4.1; 5.5]	4.9 [3.9; 5.9]	5.9 [3.0; 8.8]	4.3 [2.4; 6.2]	7.0 [5.0; 9.0]	6.2 [4.2; 8.2]	4.7 [3.6; 5.8]	5.0 [2.4; 7.6]	5.2 [3.1; 7.3]	3.4 [1.7; 5.1]	4.8 [2.7; 6.9]
Social problems	2.6 [2.1; 3.1]	2.1 [1.5; 2.7]	1.3 [0.0; 2.7]	2.2 [0.8; 3.6]	2.0 [0.9; 3.1]	2.7 [1.4; 4.0]	3.2 [2.3; 4.1]	4.8 [2.3; 7.3]	4.3 [2.4; 3.2]	1.4 [0.3; 2.5]	1.6 [0.3; 2.9]
Thought problems	6.8 [6.0; 7.6]	6.0 [4.9; 7.1]	6.3 [3.3; 9.3]	5.1 [3.0; 7.2]	7.1 [5.1; 9.1]	5.2 [3.4; 7.0]	7.9 [6.6; 9.2]	8.7 [5.3; 12.1]	7.3 [4.9; 9.7]	7.2 [4.8; 9.6]	8.4 [5.6; 11.2]
Attention problems	2.8 [2.2; 3.4]	3.0 [2.2; 3.8]	0.9 [0.0; 2.1]	5.3 [3.2; 7.4]	2.4 [1.2; 3.6]	3.5 [2.0; 5.0]	2.4 [1.7; 3.1]	2.9 [0.9; 4.9]	2.2 [0.8; 3.6]	1.4 [0.3; 2.5]	3.2 [1.4; 5.0]
Delinquent behavior	1.8 [1.4; 2.2]	1.8 [1.2; 2.4]	0.0	1.9 [0.6; 3.2]	2.3 [1.1; 3.5]	2.7 [1.4; 4.0]	1.8 [1.1; 2.5]	1.1 [0.0; 2.3]	0.8 [0.0; 1.6]	1.5 [0.4; 2.6]	4.4 [2.3; 6.5]
Aggressive behavior	0.8 [0.5; 1.1]	0.8 [0.4; 1.2]	0.0	1.1 [0.1; 2.1]	1.1 [0.3; 1.9]	1.0 [0.2; 1.8]	0.8 [0.3; 1.3]	0.4 [0.0; 1.2]	1.5 [0.4; 2.6]	0.6 [0.0; 1.3]	0.6 [0.0; 1.4]

Prevalence estimates based on German norm values; cutoffs: *T* > 63 for broadband scales (total problems, and internalizing and externalizing problems); *T* > 69 for syndrome scales

High comorbidity rates for MHP were reported in the school sample. Of the 563 adolescents scoring above the clinical cutoff in one syndrome scale, 41.2% report problems in one or more other syndrome scales (23.3% in one other scale, 8.5% in two other scales, and 9.4% in three or more other scales). Highest comorbidity rates were observed for aggressive behavior (86.7%) and anxious/depressed (86.5%). The highest combination of symptoms was reported for social problems and anxious/depressed (50%), aggressive behavior and somatic complaints (53.3%), aggressive behavior and thought problems (50.5%), and aggressive behavior and delinquent behavior (56.6%). The lowest combination of symptoms was reported for social withdrawal and delinquent (8.2%) or aggressive behavior (4.1%), somatic complaints and aggressive behavior (7.1%), anxious/depressed and aggressive behavior (9.0%), social problems and aggressive behavior (7.9%), thought problems and aggressive behavior (7.5%), and attention problems and aggressive behavior (9.7%). Comorbidities per syndrome scale are provided in Table 3.

**Table 3:** Comorbid mental health conditions of adolescents in the school sample (%)

Youth self-report syndrome scale	Social withdrawal	Somatic complaints	Anxious /depressed	Social problems	Thought problems	Attention problems	Delinquent behavior	Aggressive behavior	Any comorbid mental health condition
Social withdrawal		29.5	47.9	19.2	28.1	21.9	8.2	4.1	68.5
Somatic complaints	19.0		31.9	10.2	25.7	17.3	11.1	7.1	56.2
Anxious/depressed	45.2	46.5		24.5	37.4	26.5	12.3	9.0	86.5
Social problems	36.8	30.3	50.0		31.6	34.2	11.8	7.9	72.4
Thought problems	20.4	28.9	28.9	11.9		15.9	10.9	7.5	53.2
Attention problems	34.4	41.9	44.1	28.0	34.4		16.1	9.7	78.5
Delinquent behavior	17.6	36.8	27.9	13.2	32.4	22.1		25.0	61.8
Aggressive behavior	20.0	53.3	46.7	20.0	50.0	30.0	56.6		86.7

### Sociodemographic correlates in the school sample

The prevalence of emotional and behavioral problems for different sociodemographic characteristics is shown in Table 4. For total problems (OR = 1.66) and for internalizing problems (OR = 1.48), low SES was associated with clinically relevant scores compared to adolescents with a high SES. No differences were detected for externalizing problems. Living with a single parent was associated with a higher risk for clinically relevant problems compared to living with both biological parents (total: OR = 1.67, internalizing: OR = 1.7, externalizing: OR = 1.5). No such association was found for adolescents living in patchwork families. Migration and region were not significantly associated with YSR problem scores.

**Table 4:** Sociodemographic predictors of youth self-report (YSR) problems

Sociodemographic variable	Odds-ratio [95% confidence interval]		
	YSR total	YSR internalizing	YSR externalizing
Socioeconomic status			
High (Ref.)	1	1	1
Medium	1.12 [0.88;1.43]	1.08 [0.86;1.35]	1.17 [0.85;1.61]
Low	1.66* [1.28;2.17]	1.48* [1.08;1.91]	1.11 [0.76;1.62]
Migration status			
No (Ref.)	1	1	1
Yes	1.14 [0.93;1.40]	1.12 [0.92;1.37]	1.07 [0.80;1.42]
Family constellation			
Both parents (Ref.)	1	1	1
Single parent	1.67* [1.33;2.10]	1.70* [1.36;2.13]	1.49* [1.08;2.06]
Patchwork	1.27 [0.93;1.74]	1.13 [0.93;1.72]	1.34 [0.88;2.06]
Region			
Rural (Ref.)	1	1	1
Urban	1.04 [0.87;1.26]	1.06 [0.89;1.27]	0.94 [0.73;1.21]

*p < 0.05, *Ref*. reference category in logistic regression analysis

### Non-responder analysis

According to teachers’ ratings, adolescents that are absent from school more often (OR = 1.36, *p* < 0.001), not well integrated in class (OR = 1.38, *p* < 0.001), described as passive and withdrawn (OR = 1.15, *p* = 0.050), and described as having disciplinary problems in school (OR = 1.17, *p* = 0.040) are more likely non-responders. Furthermore, students whose parents or teaching staff have been contacted due to behavioral problems have a higher probability to be non-responders (OR = 1.26, *p* = 0.003). In contrast, female adolescents (OR = 0.83, *p* < 0.001) and adolescents who are perceived as having a better ability to concentrate during lessons (OR = 0.76, *p* < 0.001) and as making greater effort during lessons (OR = 0.76, *p* < 0.001) tend to be responders.

### Emotional and behavioral problems in the non-school sample

In the sample of unemployed adolescents, 45.9% reported internalizing and 13.5% externalizing problems (total problems: 32.4%). In the sample of child and adolescent psychiatry wards, 71.2% of adolescents reported internalizing and 27.4% externalizing problems (total problems: 64.4%), which was higher than in the school sample. The pooled total prevalence (school and non-school sample combined) is 18.2% (total problems), 19.8% (internalizing problems), and 8.1% (externalizing problems). Thus, the prevalence of MHP increased by 0.7–2.0% depending on YSR scale when the non-school sample is taken into account.

### Subthreshold DSM-5 disorders of adolescents in the school sample

The prevalence of subthreshold disorders compared to full-syndrome disorders is shown in Table 5. Besides 21.89% of adolescents suffering from any full-syndrome disorder, 12.69% are affected by any current subthreshold disorder. Subthreshold anxiety disorders were most frequent (8.1%), followed by subthreshold neurodevelopmental disorders (2.6%) and eating disorders (2.0%). Of those reporting subthreshold psychiatric disorders, 92.5% have not yet received any kind of help.

**Table 5:** Point prevalence of subthreshold DSM-5 disorders [% and (95% confidence intervals)] in the school sample compared to the prevalence of full-syndrome disorders

Psychiatric disorder	Subthreshold disorder (%)	Full-syndrome disorder (%) [6]	Subthreshold + full-syndrome disorder (%)
Neurodevelopmental disorders^a^	2.62 [2.0; 3.2]	6.50 [4.1; 8.9]	9.12 [6.3; 11.9]
Depressive disorder^b^	1.25 [0.1; 2.5]	1.36 [0.1; 2.7]	2.61 [0.9; 4.3]
Anxiety disorder^c^	8.1 [5.1; 11.1]	9.52 [6.3; 12.7]	17.62 [13.5; 21.7]
Obsessive–compulsive disorder	1.22 [0.1; 2.3]	0.70 [0.0; 1.5]	1.92 [0.5; 3.3]
Trauma- and stressor-related disorder^d^	0.78 [0.0; 1.7]	1.49 [0.3; 2.7]	2.27 [0.8; 3.7]
Eating disorder^e^	2.00 [0.5; 3.5]	0.51 [0.1; 0.9]	2.51 [0.8; 4.2]
Elimination disorder^f^	0.08 [0.0; 0.4]	0.79 [0.0; 1.7]	0.87 [0.0; 1.8]
Disruptive, impulse control, and conduct disorder^g^	0.08 [0.0; 0.4]	1.99 [0.6; 3.4]	2.07 [0.7; 3.5]
Conditions for further study			
Internet gaming disorder	0.00	0.70 [0.0; 1.5]	0.70 [0.0; 1.5]
Suicidal behavior disorder	0.26 [0.0; 0.8]	1.05 [0.1; 2.1]	1.31 [0.1; 2.5]
Nonsuicidal self-injury	0.09 [0.0; 0.4]	0.26 [0.0; 0.8]	0.35 [0.0; 1.0]
Any disorder	12.69 [9.1; 16.3]	21.89 [17.4; 26.4]	34.58 [29.4; 39.8]

^a^Including ADHD and Tic disorder

^b^Including major depression and disruptive mood dysregulation disorder

^c^Including separation anxiety disorder, selective mutism, specific phobia, social phobia, panic disorder, agoraphobia, and generalized anxiety disorder

^d^Including posttraumatic stress disorder and acute stress disorder

^e^Including anorexia nervosa, bulimia nervosa, and binge eating disorder

^f^Including enuresis and encopresis

^g^Including oppositional defiant disorder and conduct disorder

### Impairment of adolescents with subthreshold disorders

We compared adolescents who received a full-syndrome, subthreshold, and no diagnosis in the interview stage. Regarding overall HrQoL, adolescents with a subthreshold disorder (mean *t* score: 43.6; % of low HrQoL: 52%) were similarly impaired than adolescents with a full-syndrome disorder (mean *t* score: 42.0, % of low HrQoL: 48%) compared to those with no disorder (mean *t* score: 48.0, % of low HrQoL: 33%). This difference between adolescents with a subthreshold disorder and no disorder was significant (*p* < 0.05). Concerning the subcomponents of HrQoL, adolescents with a subthreshold disorder were significantly more impaired regarding the school environment compared to those with no disorder, whereas there were no significant differences for the other components. Furthermore, the self-reported school performance was significantly worse for adolescents with subthreshold disorders compared to healthy adolescents (*p* = 0.017) but similar to adolescents with full-syndrome disorders. When, additionally, asking parents, school problems were reported for 48.9% of adolescents with subthreshold disorders which is again a similar rate to those with full-syndrome disorders (57.6%) but significantly higher than for adolescents with no disorder (29.5%). Conflicts with the law were reported by 16.3% of adolescents with subthreshold disorders which was the highest rate compared to the other groups (full-syndrome disorder: 7.9%; no disorder: 6.6%). No difference between adolescents with subthreshold disorders and no disorders was observed for problems with family and peers as reported by the parents.

## Discussion

According to the current national [58, 59] and international [60] health care policies, epidemiological research is important to detect the population at need for prevention and treatment as well as to implement suitable prevention and treatment strategies to promote mental health. Especially focusing on adolescents and early interventions is named as a major concern. The purpose of this study was to determine the prevalence of MHP and associated sociodemographic correlates as well as subthreshold psychiatric disorders for a representative, large sample of Austrian adolescents to define the population at need for targeted prevention as well as to discuss different prevention strategies.

Regarding the YSR, 16.5% of the school students scored in the clinically relevant range. In accordance with the literature [12, 17, 28], approximately one out of six adolescents suffer from clinically relevant MHP. The prevalence of internalizing problems was two-to-three times higher than the prevalence of externalizing problems. These prevalence estimates increased by 0.7–2% when a non-school sample was included. MHP were often not limited to one domain. 41.2% scored in the clinically relevant range in more than one syndrome scale of the YSR. A low SES and living with single parents turned out as significant risk factors. Structured clinical interviews revealed a prevalence of 12.7% for any subthreshold DSM-5 disorder, with subthreshold anxiety disorders being the most frequent.

The YSR total mean problem score of this study (34.2) is in the mid-range compared to mean scores observed in other European societies (grand mean = 35.3, range = 25.0–49.3 [29]) with mainly northern countries (e.g., Germany, Finland, Norway, Netherlands, and Denmark) reporting lower mean scores and mainly southern countries (e.g., Portugal, Spain, Greece, and Italy) reporting higher mean scores. However, these comparisons only allow an approximate classification of the Austrian mean problem score compared to other European samples as the methodology of the studies varies regarding the included age range and the sampling frame.

In our study as in other studies using the YSR [29], internalizing problems were reported more often (17.8%) than externalizing problems (7.4%).

In detail, somatic complaints, thought problems, social withdrawal, and anxious/depressed were most prevalent; attention problems, social problems, aggressive behavior, and delinquent behavior were least prevalent, with 41.2% reporting problems in more than one syndrome scale being in accordance with recent research [1, 10, 17]. Highest comorbidity is shown for aggressive behavior (in combination with somatic complaints, thought problems, and delinquent problems) and anxious/depressed symptoms (in combination with social withdrawal and somatic complaints). The fact that MHP are often not limited to one domain but co-occur with others raises important implications for prevention programs. The majority of prevention programs for adolescents’ target single mental health domains, e.g., anxiety or aggression only [26, 61, 62]. As comorbidity rates are high (more than 50%) for all of the syndrome scales, addressing only one single symptom does not seem enough for prevention programs. Our results indicate the need for prevention programs that consider a broad range of comorbidities. In accordance with our results, such combined or expanded targeted prevention programs could address anxious and depressive symptoms combined with social or somatic problems as well as aggression and delinquent problems with thought or somatic problems, e.g., aggressive behavior has the highest percentage of any comorbid mental health condition, leading to the assumption that all of the other affected domains should also be addressed in prevention programs targeting aggression.

Consistent with the literature [29], girls reported more total and internalizing problems than boys. There was no difference for externalizing problems, differing from other findings stating more problems for boys [2, 29]. Externalizing problems increased by age for girls and boys, whereas total and internalizing problems did not. This result diverges from findings about older adolescents reporting more total [29] and internalizing problems [1].This may be because we only included adolescents from 11 years onwards and internalizing problems are known to increase from childhood to adolescence [2, 10]. However, the fact that younger adolescents are equally affected by internalizing problems than older adolescents emphasizes the need for the early prevention.

Our results concerning sociodemographic correlates are largely consistent with the literature. As often reported [9, 11, 12, 30, 31], low SES was associated with a higher risk for internalizing, but not for externalizing problems. In contrast to other studies [31–33], we found no association with migration status. However, as non-German speakers have been excluded, the sample of adolescents reporting migration background may not be representative, which is a known problem in epidemiological research [12]. Our results confirm that adolescents living with both parents report less problems [9, 10, 31, 36, 37], underlying the impact of non-intact homes on MHP. There was no influence of region, in contrast to the literature [9, 31] but being consistent with Merikangas et al. [10]. However, these findings reveal the need for prevention targeting adolescents from disadvantaged and non-intact families.

Important implications can be drawn from our non-responder analysis. Non-response was associated with higher school absenteeism, worse integration in class, more withdrawn behavior and disciplinary problems, and contacting parents or school staff due to a behavioral problem, whereas responders were perceived as having a better ability to concentrate and making greater effort during lessons. Despite effect sizes were minimal to low, responders differ from non-responders regarding school-related problems. This indicates an underestimation of obtained prevalence estimates, as also discussed in the previous epidemiological studies [39, 40]. Including non-responder analyses in epidemiological studies is, therefore, all the more important to evaluate a potential bias and to draw reasonable conclusions regarding the validity of prevalence estimates.

We additionally obtained a non-school sample of adolescents who are hard to reach and, therefore, often not approached in epidemiological studies [41] including samples from child and adolescent psychiatry wards and courses for unemployed adolescents. Including hard-to-reach samples helps in enhancing the generalizability of results and overcoming the limitations of using a school sample only. As the prevalence of unemployed adolescents scoring in the clinically relevant range was more than double as high as in the school sample, our results demonstrate that this population is at special risk for MHP and represents a special target group for prevention programs.

In addition to the data on MHP derived from the YSR, rates of subthreshold psychiatric disorders provide extra information about the population needing prevention. Compared to about 22% of adolescents diagnosed with full-syndrome disorders needing specialized treatment, about 13% of adolescents report subthreshold disorders, with 92.5% of them receiving no help. Subthreshold anxiety disorders were most often diagnosed. For obsessive–compulsive disorders and eating disorders, there was an even higher rate of subthreshold than full-syndrome disorders. Although this is a cross-sectional study and no predictions can be made if adolescents with subthreshold disorders will develop a full-syndrome disorder or if symptoms will vanish over time without any intervention, the findings on HrQoL indicate that this group face similar psychosocial impairments in some domains of HrQoL as adolescents with full-syndrome disorders. Thus, targeted prevention should be provided to prevent symptom progression.

Due to compulsory schooling, school-based approaches seem suitable for prevention programs as school is a setting where such programs can reach all children and young adolescents [60]. Universal prevention can be combined with targeted prevention programs as the latter is known to be more effective [26]. Furthermore, after screening for various MHP, prevention programs can be tailored based on the individual risk profile of adolescents. Such approaches already exist for instance for eating disorders [63]. Based on a screening, students are either directed to a universal prevention program (if there are no signs at all for disordered eating behavior), a targeted prevention program (for individuals at risk for an eating disorder), or a treatment (if they already show clinically relevant signs of an eating disorder). Similar approaches are conceivable for several disorders. We would suggest a stepwise, combined approach, offering universal prevention for adolescents neither at risk nor showing subthreshold symptoms, selective prevention for high-risk groups (like low socioeconomic status, etc.), indicated prevention for adolescents at risk (using a screening questionnaire like the YSR) or adolescents suffering from subthreshold disorders (showing subclinical symptoms) or treatment for adolescents fulfilling full-syndrome disorders. Such a program should provide different modules for all possible combinations of symptoms. The European initiative “ICare” (http://www.icare-online.eu) is currently working on establishing an online platform incorporating a comprehensive online screening and different online interventions for common MHP. Based on the screening results, participants will be automatically directed to an online prevention or intervention program that fits the participant’s risk status and symptoms.

Besides using a school approach, courses for unemployed adolescents seem to be a potential setting to reach at-risk populations. Furthermore, adolescents seeking help at psychiatric clinics because of diagnosed disorders may benefit from targeted prevention of known comorbid conditions. In addition, as having a family member with a mental illness is known as risk factor [64], the whole family of a person suffering from a psychiatric disorder should be introduced to targeted prevention programs for relatives immediately when the effected person first contacts the health care system. A broader public health approach through nonprofit organizations or counseling centers which may be the first stop for people with MHP seems conceivable. Furthermore, different help lines could lead help seeking adolescents to targeted prevention programs. Then, in times of increased Internet usage, a web-based approach should be taken into account, detecting high-risk populations through online counseling or self-help websites. Finally, it may also be promising to detect at-risk adolescents through social media (e.g., content analysis or hashtag searches) and offer targeted prevention programs.

### Limitations

One limitation is the high rate of non-responders, which usually affects studies based on voluntary samples and often leads to an underestimation of MHP [40]. There was a high non-response of schools, caused by rejections through headmasters, leaving 16.8% of all schools for sampling procedure. Only 45.1% of adolescents of the stratified sample responded. Another limitation is the exclusion of non-German speakers, which may lead to an underrepresentation of adolescents with migration background. Recent migrants not yet attending any school, inpatient adolescents for medical reasons, and homeless adolescents not attending a school were also not included. Because of the cross-sectional design, associations between sociodemographic variables and MHP can only be interpreted in terms of correlations and not causally. Concerning MHP derived from the questionnaire, only data from adolescents but not from their parents were derived.

### Strengths

This is the first study obtaining MHP and subthreshold disorders in a representative national sample of Austrian adolescents. A pilot study was conducted to evaluate feasibility and acceptability from teachers’ perspective. As recommended for epidemiological studies [39, 40], a non-responder analysis was conducted, which was not limited to sociodemographic variables only but included the assessment of school-related problems that can be indicative of MHP, which is of importance to evaluate a potential non-response bias. Furthermore, a non-school sample was obtained to include adolescents that are hard to reach, which is quite a new aspect in epidemiological research based on school samples. Estimations based on school samples only may underestimate the prevalence of MHP. The importance of including hard-to-reach samples in epidemiological studies is strongly supported by the fact that the early school leavers had a prevalence of MHP that was about double as high as in students. This should encourage researchers for future epidemiological studies. Finally, reported subthreshold disorders were taken into account to underline the importance of prevention strategies in addition to specific therapeutic approaches to prevent the development of full-syndrome disorders.

## Conclusion

A significant proportion of adolescents is at risk for MHP and show subthreshold psychiatric disorders. The non-responder analysis indicates that the prevalence may even be underestimated. The findings of the present study emphasize the urgent need for targeted prevention. Possible comorbid MHP should clearly be taken into account. Adolescents in disadvantaged families and early school leavers are a special target group for prevention programs. Furthermore, strategies to enhance help seeking behavior in at-risk adolescents should be developed. Altogether, being in line with national and international health care policies, our results emphasize the significance of implementing prevention strategies for children and adolescents to improve their well-being, reduce symptom progression and development of full-syndrome disorders, and, therefore, reduce costs for the health care system.
